# Unraveling the Molecular Mechanisms of Blueberry Root Drought Tolerance Through Yeast Functional Screening and Metabolomic Profiling

**DOI:** 10.3390/plants13243528

**Published:** 2024-12-17

**Authors:** Xinyu Fan, Beijia Lin, Yahong Yin, Yu Zong, Yongqiang Li, Youyin Zhu, Weidong Guo

**Affiliations:** 1 College of Life Sciences, Zhejiang Normal University, Jinhua 321004, China; fanxinyu2000@zjnu.edu.cn (X.F.); linbeijia@zjnu.edu.cn (B.L.); yinyahong@zjnu.edu.cn (Y.Y.); yzong@zjnu.cn (Y.Z.); lyq@zjnu.cn (Y.L.); 2 Zhejiang Provincial Key Laboratory of Biotechnology on Specialty Economic Plants, Zhejiang Normal University, Jinhua 321004, China; 3 College of Agricultural, Jinhua University of Vocational Technology, Jinhua 321007, China

**Keywords:** blueberry root, drought tolerance, molecular mechanisms, yeast functional screening, genes, metabolomic profiling

## Abstract

Blueberry plants are among the most important fruit-bearing shrubs, but they have shallow, hairless roots that are not conducive to water and nutrient uptake, especially under drought conditions. Therefore, the mechanism underlying blueberry root drought tolerance should be clarified. Hence, we established a yeast expression library comprising blueberry genes associated with root responses to drought stress. High-throughput sequencing technology enabled the identification of 1475 genes potentially related to drought tolerance. A subsequent KEGG enrichment analysis revealed 77 key genes associated with six pathways: carbon and energy metabolism, biosynthesis of secondary metabolites, nucleotide and amino acid metabolism, genetic information processing, signal transduction, and material transport and catabolism. Metabolomic profiling of drought-tolerant yeast strains under drought conditions detected 1749 differentially abundant metabolites (DAMs), including several up-regulated metabolites (organic acids, amino acids and derivatives, alkaloids, and phenylpropanoids). An integrative analysis indicated that genes encoding several enzymes, including *GALM*, *PK*, *PGLS*, and *PIP5K*, modulate key carbon metabolism-related metabolites, including D-glucose 6-phosphate and β-D-fructose 6-phosphate. Additionally, genes encoding *FDPS* and *CCR* were implicated in terpenoid and phenylalanine biosynthesis, which affected metabolite contents (e.g., farnesylcysteine and tyrosine). Furthermore, genes for *GST* and *GLT1*, along with eight DAMs, including L-γ-glutamylcysteine and L-ornithine, contributed to amino acid metabolism, while genes encoding *NDPK* and *APRT* were linked to purine metabolism, thereby affecting certain metabolites (e.g., inosine and 3′,5′-cyclic GMP). Overall, the yeast functional screening system used in this study effectively identified genes and metabolites influencing blueberry root drought tolerance, offering new insights into the associated molecular mechanisms.

## 1. Introduction

Global warming exacerbates drought stress, which poses a significant threat to the growth and development of plants, including fruit trees that are often cultivated on mountainous lands with low water-retention capacity [1]. The resulting water scarcity can diminish fruit quality and yield, disrupt physiological processes, and alter fruit tree growth and development [2]. Hence, elucidating the mechanisms by which fruit trees respond to drought stress is essential. Roots, being in direct contact with the soil and responsible for water absorption, are the first plant parts to be affected by soil water deficits. Plant roots have evolved various adaptive mechanisms that mitigate drought-induced damage by altering root structures, regulating osmotic pressure, and activating the antioxidant system [3], often in concert.

Plant metabolites, which are small molecules and intermediates produced during metabolism, are crucial for growth, reproduction, and environmental responses. Under drought conditions, metabolic networks are significantly reconfigured, leading to the synthesis of several metabolites, including organic acids [4], betaine [5], flavonoids [6], proline [7], and soluble sugars [8], which enhance drought tolerance by adjusting osmotic pressure and stabilizing membrane structures. Researchers have focused on identifying these metabolites and characterizing their regulatory mechanisms, typically through integrated transcriptome and metabolome analyses [9]. This approach has resulted in the identification of many key regulatory genes and metabolites involved in specific biological pathways or responses [10,11,12].

This integrated analysis involves inherent biological processes. During stress responses, the expression of specific genes is induced, with the encoded proteins regulating metabolic pathways and promoting metabolite synthesis. The metabolites are then analyzed using ultra-high performance liquid chromatography (UHPLC) systems [13]. However, to accurately determine gene functions, additional biotechnological methods are required. A technical system that screens for and identifies metabolites and the associated regulatory genes can enhance research efficiency. Yeast (*Saccharomyces cerevisiae*), with its simple culture requirements, rapid growth, and well-defined genetics, can be used to clone and functionally characterize plant genes under stress conditions [14]. This approach, which involves a yeast-based rapid screening system (YRSS), has been widely used to identify plant gene functions under stress [15].

Blueberry (*Vaccinium* spp.) is a perennial shrub widely cultivated for its nutritious fruits, but the global production of blueberry fruits is significantly threatened by drought stress [16]. In China, blueberry plants are commonly grown in the northern region, but they are now also cultivated in the southern region, where they grow faster and their fruits ripen earlier, despite facing certain environmental challenges (e.g., high temperatures and increased drought risk). The shallow, hairless roots of blueberry plants have adapted to environmental fluctuations, including decreases in water availability [16,17], underscoring the importance of clarifying drought tolerance mechanisms for breeding more resilient cultivars.

Despite numerous studies on blueberry drought tolerance, a comprehensive assessment of the root system response to drought is lacking, which has limited our understanding of drought tolerance mechanisms [16]. Additionally, the challenges of genetic transformation in non-model plants, such as blueberry, have hindered the verification of gene functions. Employing YRSS may expedite blueberry drought tolerance research. In this study, we simulated drought stress using PEG-6000, extracted mRNA from treated roots, reverse-transcribed mRNA to cDNA, and constructed expression vectors that were introduced into yeast cells to create an expression library. After rigorously screening yeast cells for drought tolerance, we selected the surviving yeast cells for high-throughput plasmid and metabolite assays. Our integrative analysis identified key signaling pathways contributing to the blueberry root drought response, thereby establishing a comprehensive YRSS to elucidate blueberry drought tolerance mechanisms and identify molecular targets for improving drought tolerance.

## 2. Results

### 2.1. High PEG-6000 Concentrations Affect Blueberry Growth

**Figure 1:**
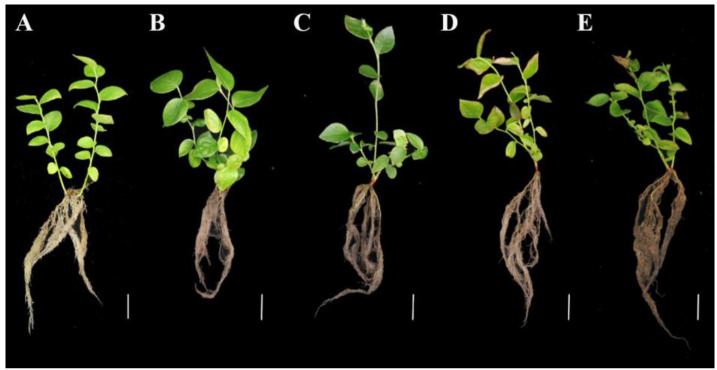
Blueberry plant growth changes following treatments with different PEG-6000 concentrations. (**A**) Control; (**B**) 5% PEG-6000 treatment for 48 h; (**C**) 10% PEG-6000 treatment for 48 h; (**D**) 15% PEG-6000 treatment for 48 h; (**E**) 20% PEG-6000 treatment for 48 h. Bar: 2 cm.

**Figure 2:**
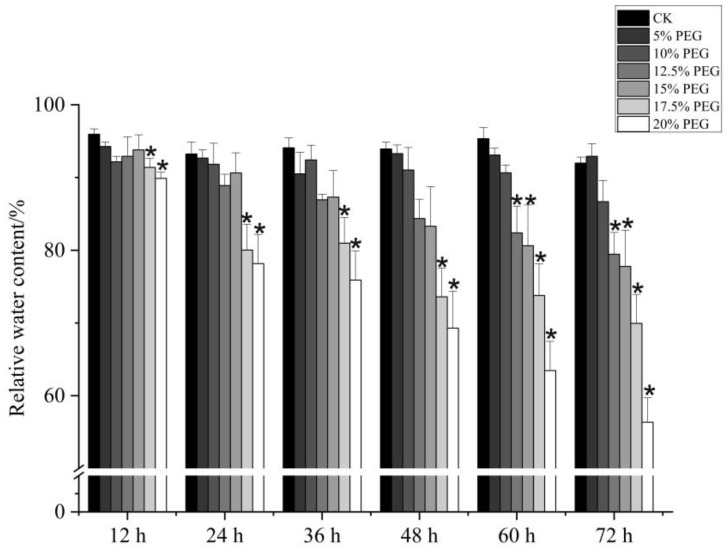
Trends in the changes in the relative water content of ‘Emerald’ blueberry leaves after treatments with different PEG-6000 concentrations. * indicates a significant difference (*p* < 0.05) relative to the control group.

To assess the effect of drought on blueberry plant growth, PEG-6000 was used to simulate drought conditions that lead to osmotic stress. ‘Emerald’ blueberry cuttings were immersed in half-strength Hoagland nutrient solution containing varying PEG-6000 concentrations for 72 h. Relative to the control (Figure 1A), low PEG-6000 concentrations (5% and 10%) did not produce noticeable effects (Figure 1B,C). In contrast, higher PEG-6000 concentrations (12.5%, 15%, 17.5%, and 20%) significantly affected blueberry root health and the leaf relative water content (RWC). Specifically, the 15% PEG-6000 treatment led to root browning as well as leaf curling and desiccation (Figure 1D), whereas the 20% PEG-6000 treatment resulted in considerable root browning and leaf wrinkling (Figure 1E). The leaf RWC decreased by 11.3% and 26.2% following the 15% and 20% PEG-6000 treatments, respectively (Figure 2). These findings indicate that high PEG-6000 concentrations can simulate severe drought conditions, leading to diminished water absorption by roots. However, blueberry cuttings can withstand the mild osmotic stress induced by lower PEG concentrations. For this study, a 72 h exposure to PEG-6000 was determined to be an adequate treatment for inducing a clear drought stress response in blueberry roots.

### 2.2. Construction of a Yeast Expression System and Drought Tolerance Screening

To construct a yeast expression library enriched with genes related to blueberry drought tolerance, we initially created an *Escherichia coli* library. Total RNA was isolated from blueberry roots treated with PEG-6000, after which cDNA was synthesized and amplified via PCR. After purification, the cDNA was cloned into the pYES2 vector for the subsequent transformation of *E. coli* cells to establish the cDNA library. Quantitative assessment revealed the library capacity was approximately 2.456 × 10^8^ and the total clone count was roughly 4.912 × 10^8^ (Figure S1A). Randomly selected clones were analyzed by PCR to determine insertion lengths, which ranged from 0.5 to > 2 kb (Figure S1B). Thus, the library was successfully created. Recombinant plasmids were then inserted into yeast strain *ycf1* cells. According to a PCR analysis of 24 randomly selected yeast clones, the fragment sizes were as expected, and a titer of 3.36 × 10^8^ cfu/mL was obtained, reflecting the high quality of the yeast expression system (Figure S2).

**Figure 3:**
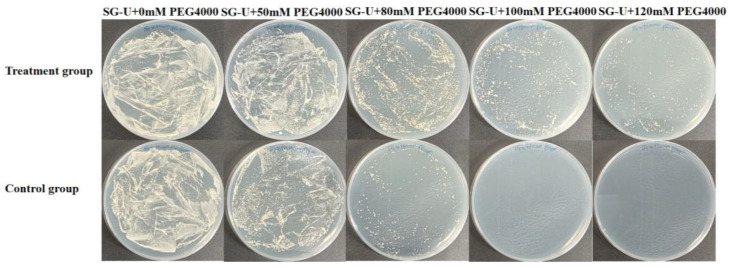
Yeast library was treated with different PEG-4000 concentrations (0, 50, 80, 100, and 120 mM) to select the appropriate simulated drought concentration.

Functional genes were identified by screening with PEG-4000. The optimal working concentration was established after observing that some working group (WG) cells proliferated on medium supplemented with 100 mM PEG-4000, whereas all control group (CG) cells failed to grow (Figure 3). This difference in osmotic stress tolerance between WG and CG cells at 100 mM PEG-4000 suggested that the enhanced tolerance of WG cells was due to the presence of blueberry-derived drought tolerance-related genes that support critical cellular functions and growth under osmotic stress. Consequently, 100 mM PEG-4000 was selected for screening the yeast library. The remaining viable yeast cells were collected and examined to identify drought tolerance-related gene sequences.

After PEG-tolerant clones were selected and the drought stress library was produced, the library inserts were amplified by PCR and sequenced using Illumina technology. For the analysis of sequencing data, ‘read count’ refers to the number of sequencing reads that were aligned with a given gene, with higher read counts indicating a greater likelihood that the gene is involved in drought tolerance mechanisms. In this study, genes with read counts of at least 20 were considered as potential candidate genes mediating drought tolerance. A total of 1475 genes were identified as potential drought stress response-related genes (Table S2).

### 2.3. Gene Ontology (GO) Annotation of Genes in the Drought Stress Tolerance Library

**Figure 4:**
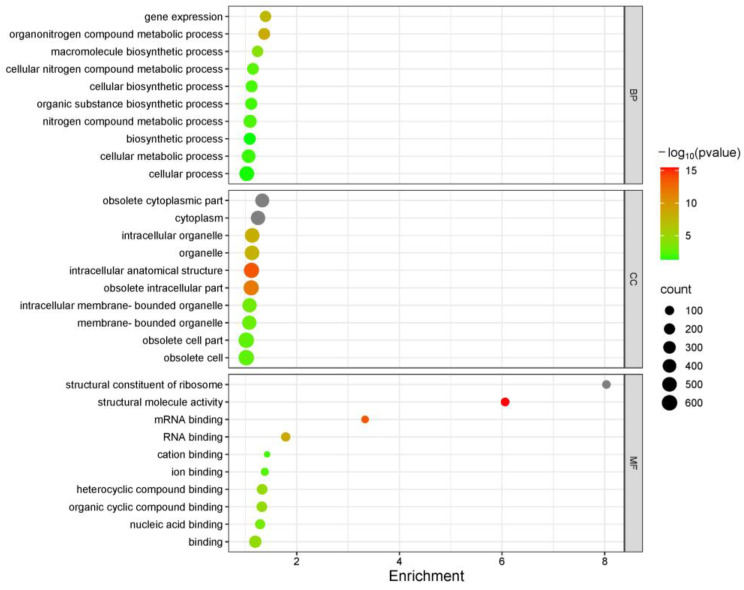
Top 10 GO terms assigned to drought tolerance-related genes.

Genes involved in the blueberry root response to drought stress were identified on the basis of a GO enrichment analysis that functionally annotated genes. The drought-responsive genes were annotated with 548 GO terms from the three main GO categories. The 10 most significantly enriched GO terms in each main category are presented in Figure 4. In the biological process category, 529 genes (i.e., 35.86% of all genes) were associated with ‘cellular process’ (GO:0009987); the GO terms assigned to these genes included ‘cellular metabolic process’ (GO:0044237), ‘cellular biosynthetic process’ (GO:0044249), and ‘cellular nitrogen compound metabolic process’ (GO:0034641). The main GO terms assigned to the genes related to metabolic processes were ‘nitrogen compound metabolic process’ (GO:0006807) and ‘organonitrogen compound metabolic process’ (GO:1901564); these two GO terms were assigned to 40.68% of the genes. These findings imply cellular and metabolic processes enhance blueberry root drought tolerance. In the cellular component category, numerous genes (32.27% of all genes) were associated with ‘intracellular membrane-bounded organelle’ (GO:0043231), suggesting that osmoregulation in cellular compartments may contribute to drought tolerance. In the molecular function category, ‘binding’ (GO:0005488) was the most enriched term, accounting for 18.98% of the genes. Other enriched binding-related GO terms included ‘heterocyclic compound binding’ (GO:1901363), ‘RNA binding’ (GO:0003723), and ‘ion binding’ (GO:0043167). This enrichment underscores the importance of these binding functions for the mechanism mediating blueberry root drought tolerance.

### 2.4. Kyoto Encyclopedia of Genes and Genomes (KEGG) Enrichment Analysis of Genes in the Drought Stress Tolerance Library

**Figure 5:**
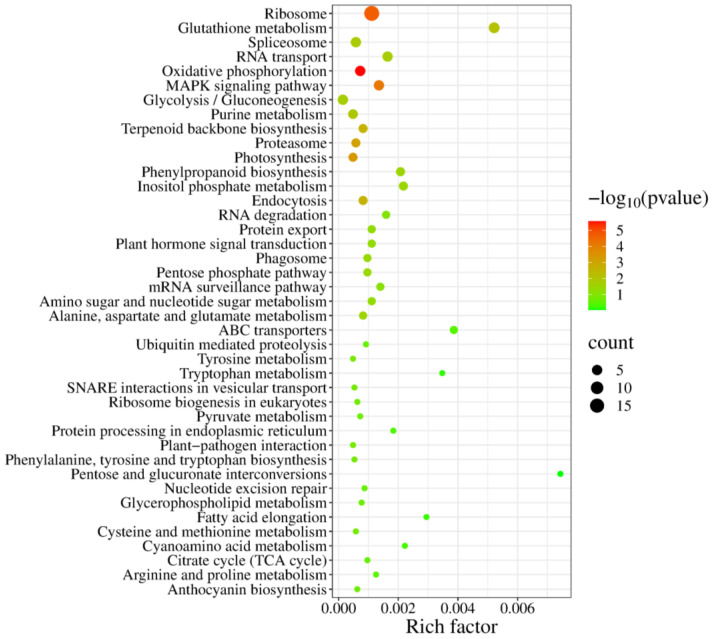
Enriched KEGG pathways among drought tolerance-related genes. The dot area represents the relative number of isolated genes in the pathway, whereas the dot color represents the Q value.

According to the significantly enriched KEGG pathways (*p* < 0.05) (Figure 5), drought tolerance-related genes were divided into the following six functional groups (Table S3): carbon and energy metabolism (Group 1), biosynthesis of secondary metabolites (Group 2), nucleotide and amino acid metabolism (Group 3), genetic information processing (Group 4), signal transduction (Group 5), and material transport and catabolism (Group 6). Group 1 contained 10 genes participating in carbon metabolism, including two aldose 1-epimerase (*GALM*) genes and three pyruvate kinase (*PK*) genes associated with the glycolysis/gluconeogenesis pathway, two 6-phosphogluconolactonase (*PGLS*) genes involved in the pentose phosphate pathway, and three 1-phosphatidylinositol-4-phosphate 5-kinase (*PIP5K*) genes contributing to inositol phosphate metabolism. This group also included five genes encoding the gamma subunit of F-type H^+^-ATPase and three genes encoding ferredoxin, all of which have integral roles in energy metabolism.

Group 2 included three farnesyl diphosphate synthase (*FDPS*) genes essential for terpenoid backbone biosynthesis and three genes encoding cinnamoyl-CoA reductase (*CCR*), which functions in the phenylpropane biosynthesis pathway. Group 3 consisted of several drought tolerance-related genes, including two genes encoding nucleoside-diphosphate kinase (*NDPK*) and two genes encoding adenine phosphoribosyl transferase (*APRT*), which are related to purine metabolism. Six genes encoding glutathione S-transferase (*GST*), which is crucial for mercapturic acid synthesis, were associated with glutathione metabolism. Additionally, among the genes mediating alanine, aspartate, and glutamate metabolism, two encoded glutamate synthase (*GLT1*), which is required for glutamate production.

In Group 4, the genetic information processing genes included 18 genes encoding ribosomal proteins, including the large-subunit proteins L23e, L24e, L28, and L34e as well as the small-subunit proteins S14e and S19e. This group also contained three genes encoding the 26S proteasome regulatory subunit N11, three genes for U1 small nuclear ribonucleoprotein C, and two genes for the splicing factor U2AF 65 kDa subunit. Of the genes related to RNA functions, three and two were associated with the small ubiquitin-related modifier SUMO and the elongation factor EF1A, respectively.

Group 5 included five genes encoding CaM family members involved in calcium signaling pathways. Group 6 contained three genes related to endocytosis (*RABF2A* and *ARA7*) and two genes related to phagolysosomes (*TUA2*), highlighting their potential role in enhancing blueberry root tolerance to drought stress.

### 2.5. Candidate Genes That Significantly Enhance Yeast Drought Tolerance

**Figure 6:**
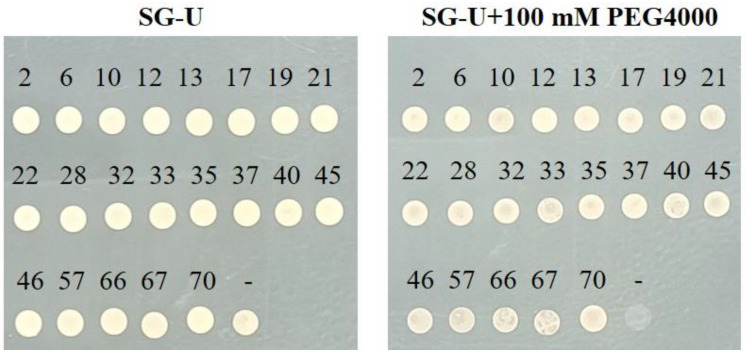
Verification of drought-tolerant yeast clones. The number above is the number of positive yeast clones.

To evaluate whether the identified candidate genes confer drought tolerance, 96 randomly selected yeast colonies were analyzed. Initially, a PCR amplification was performed to isolate blueberry genes in yeast colony cells. The amplified products were sequenced and functionally annotated, leading to the identification of 21 drought tolerance-related candidate genes. Subsequently, pYES2 vectors harboring these 21 genes were inserted into *ycf1* cells to generate transformed yeast cells expressing blueberry genes. Both transformed and non-transformed yeast cells were used to inoculate media with or without PEG-4000. After a 3-day incubation, yeast cells transformed with one of the 21 genes grew normally on both media, whereas the non-transformed yeast cells grew only on the medium lacking PEG-4000 (Figure 6). Accordingly, these 21 genes appeared to enhance the tolerance of yeast cells to PEG-4000. Thus, we speculated that the expression of these genes may improve the drought tolerance of blueberry roots. Furthermore, these findings reflect the feasibility and practicality of YRSS for the large-scale identification of drought tolerance-related genes in blueberry roots.

### 2.6. Identification and Analysis of Differentially Abundant Metabolites (DAMs)

**Figure 7:**
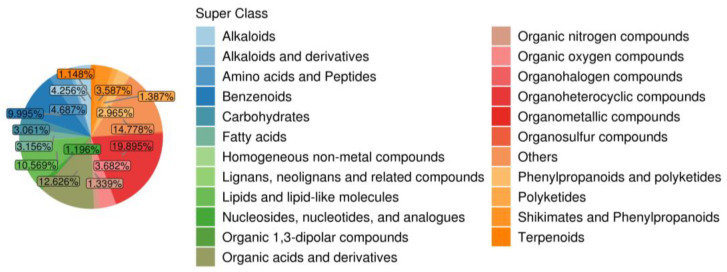
Metabolite classifications and proportions.

In this study, we investigated the metabolic response of blueberry to drought stress by conducting a UHPLC-MS-based metabolomic analysis. We identified 2091 metabolites, which were classified into 23 major groups, including amino acids and derivatives (4.687%), lipids and lipid-like molecules (10.569%), organic acids and derivatives (12.626%), alkaloids (4.256%), benzenoids (9.995%), phenylpropanoids and polyketides (2.965%), and others (Figure 7). According to a principal component analysis (PCA), the samples were clearly separated into three clusters along the first principal component (PC1) and were tightly grouped within each cluster along the second principal component (PC2), indicative of the accuracy of the model (Figure S3). An orthogonal partial least squares discriminant analysis (OPLS-DA) further validated the model, with R^2^Y and Q^2^ values exceeding 0.5, indicating reliability and stability (Figure S4).

**Figure 8:**
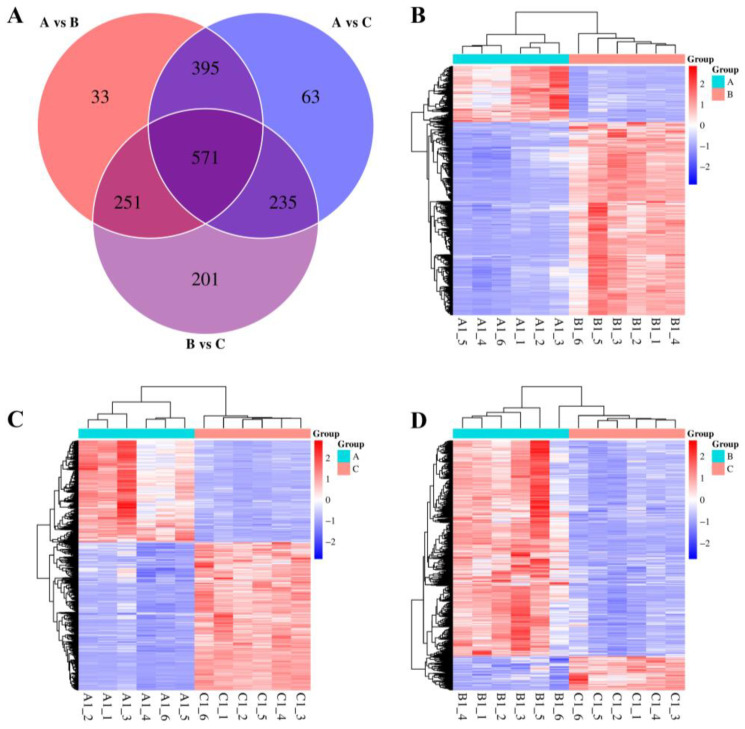
Overview of the identified DAMs. (**A**) Venn diagram of the results of the comparisons of three groups (i.e., A, B, and C); (**B**–**D**) Heat maps of DAMs between different groups.

DAMs were determined on the basis of VIP > 1.0 and *p* < 0.05 (Figure S5). Of the 1749 significant DAMs, 571 were common to all three group comparisons. These DAMs were mainly organic acids, amino acids and peptides, and phenylpropanoids (Figure 8A). A fold-change analysis identified 969 up-regulated and 281 down-regulated DAMs between Groups A and B. The up-regulated DAMs were terpenoids, shikimates and phenylpropanoids, lipids and lipid-like molecules, and organic acids and derivatives (Figure 8B), suggesting that the introduction of exogenous blueberry genes significantly affected the yeast metabolite profile. The comparison of Groups A and C yielded 749 up-regulated and 515 down-regulated DAMs, with terpenoids, lipids and lipid-like molecules, alkaloids, and shikimates and phenylpropanoids detected as the most up-regulated metabolites (Figure 8C), implying that the expression of exogenous blueberry genes enhances yeast drought tolerance via increased metabolite synthesis. The comparison of Groups B and C revealed 171 up-regulated and 1087 down-regulated DAMs, with terpenoids, organic acids and derivatives, phenylpropanoids, and alkaloids among the up-regulated metabolites (Figure 8D). The substantial number of down-regulated DAMs in Group C suggests that drought stress resulted in significant yeast cell mortality, but metabolites in yeast cells expressing blueberry genes may play a key role in the mechanism underlying drought tolerance. Collectively, these findings indicate that the adaptation of blueberry roots to drought involves changes to metabolite levels.

### 2.7. Carbohydrate Metabolism and Its Association with Blueberry Drought Tolerance

**Figure 9:**
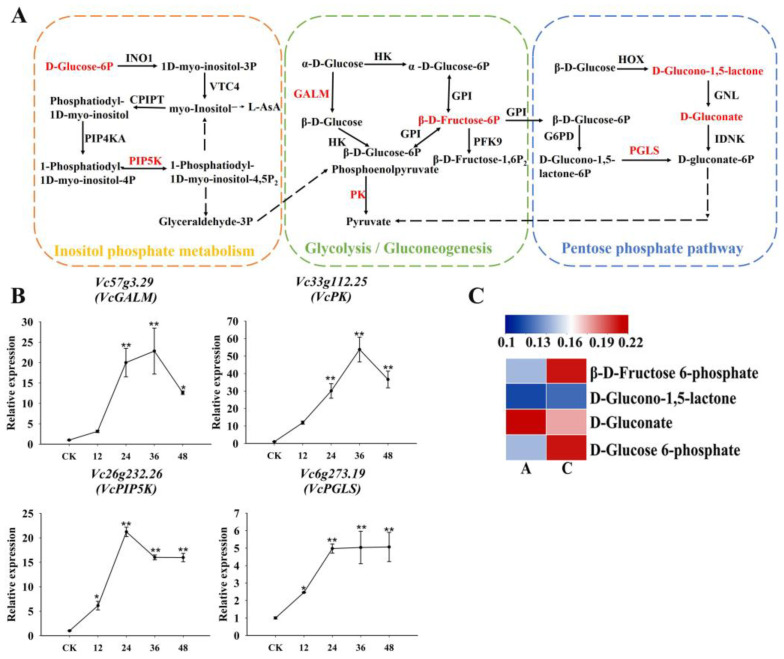
Association analysis of drought tolerance-related genes and DAMs in carbohydrate metabolism pathways. (**A**) Inositol phosphate metabolism, glycolysis/gluconeogenesis, and pentose phosphate pathway; (**B**) qRT-PCR results for four drought tolerance-related genes involved in carbohydrate metabolism; (**C**) Heat map of DAMs involved in carbohydrate metabolism. Colors reflect the regulation of metabolites under drought conditions (indicated in the scale bar). * and ** represented significant difference under *p* < 0.05 and *p* < 0.01, respectively.

In the carbon metabolism pathway (Figure 9A), genes encoding four enzymes (*GALM*, *PK*, *PGLS*, and *PIP5K*) as well as four DAMs (D-glucose 6-phosphate, β-D-fructose 6-phosphate, D-glucono-1,5-lactone, and D-gluconate) were identified. According to quantitative real-time polymerase chain reaction (qRT-PCR) data, the expression of these genes tended to increase after the 15% PEG-6000 treatment (Figure 9B). In addition, the D-glucose 6-phosphate, β-D-fructose 6-phosphate, and D-glucono-1,5-lactone contents were significantly higher in Group C than in Group A under drought conditions (Figure 9C).

### 2.8. Involvement of Terpenoid and Phenylpropanoid Biosynthesis in the Regulation of Blueberry Root Drought Tolerance

**Figure 10:**
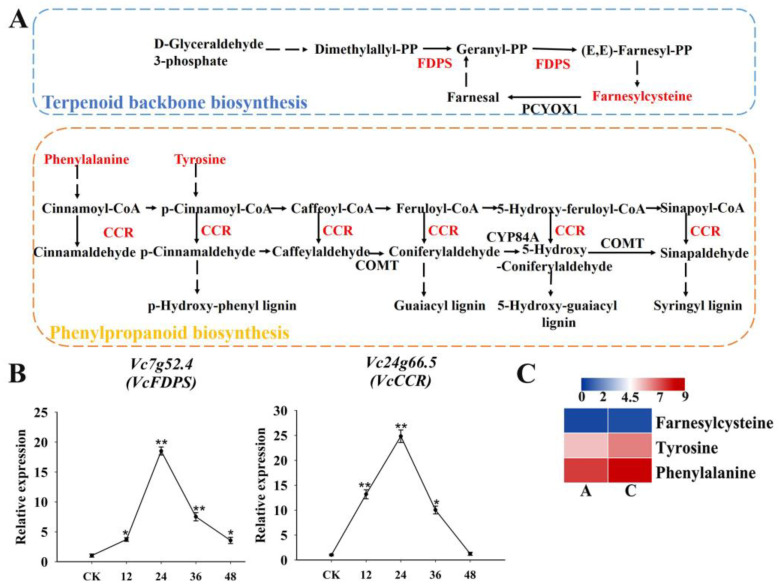
Association analysis of drought tolerance-related genes and DAMs in secondary metabolite biosynthesis pathways. (**A**) Terpenoid backbone biosynthesis pathway and phenylpropanoid biosynthesis pathway; (**B**) qRT-PCR results for two drought tolerance-related genes involved in secondary metabolite biosynthesis; (**C**) Heat map of DAMs involved in secondary metabolite biosynthesis. Colors reflect the regulation of metabolites under drought conditions (indicated in the scale bar). * and ** represented significant difference under *p* < 0.05 and *p* < 0.01, respectively.

Multiple genes associated with terpenoid and phenylpropanoid biosynthesis pathways were identified. In the terpenoid backbone biosynthesis pathway (Figure 10A), one *FDPS* gene mediated terpenoid biosynthesis and promoted the accumulation of farnesylcysteine under drought stress (Figure 10B, C). In the phenylpropanoid biosynthesis pathway (Figure 10A), the *CCR* gene expression level increased in response to drought stress (Figure 10B), leading to the accumulation of two DAMs (tyrosine and phenylalanine) (Figure 10C).

### 2.9. Enrichment of Drought Tolerance-Related Genes in the Amino Acid and Nucleotide Metabolism Pathways

**Figure 11:**
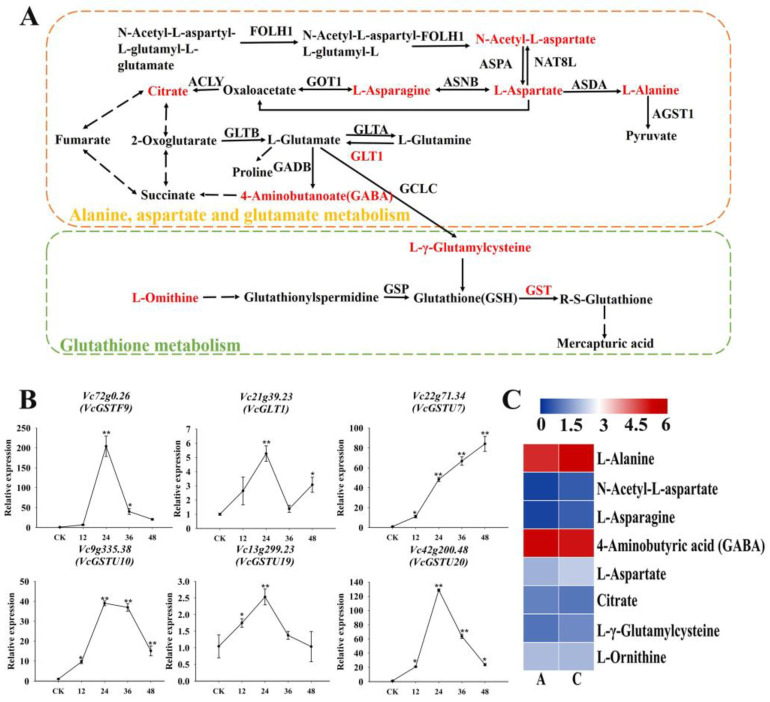
Association analysis of drought tolerance-related genes and DAMs in amino acid metabolism pathways. (**A**) Alanine, aspartate, and glutamate metabolism and glutathione metabolism; (**B**) qRT-PCR results for six drought tolerance-related genes involved in amino acid metabolism; (**C**) Heat map of DAMs involved in amino acid metabolism. Colors reflect the regulation of metabolites under drought conditions (indicated in the scale bar). * and ** represented significant difference under *p* < 0.05 and *p* < 0.01, respectively.

**Figure 12:**
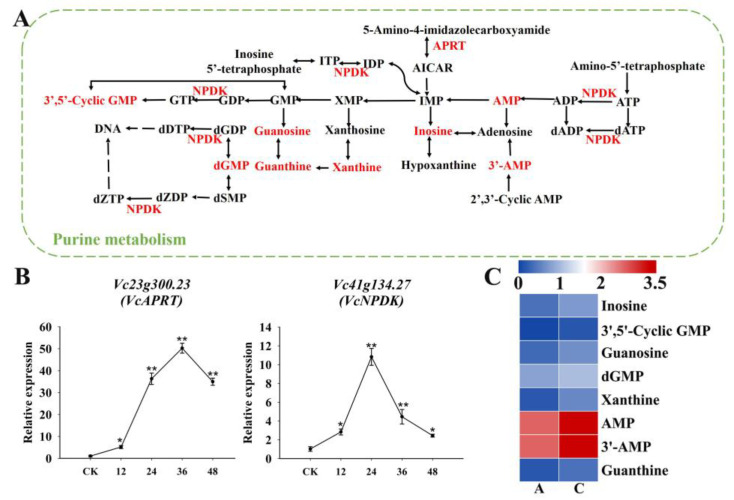
Association analysis of drought tolerance-related genes and DAMs in a nucleotide metabolism pathway. (**A**) Purine metabolism; (**B**) qRT-PCR results for six drought tolerance-related genes involved in nucleotide metabolism; (**C**) Heat map of DAMs involved in nucleotide metabolism. Colors reflect the regulation of metabolites under drought conditions (indicated in the scale bar). * and ** represented significant difference under *p* < 0.05 and *p* < 0.01, respectively.

Among the identified genes in the amino acid metabolism pathway (Figure 11A), the expression levels of glutathione S-transferase (*GST*) and glutamate synthase (*GLT1*) genes were up-regulated following the drought treatment (Figure 11B), which promoted the accumulation of the following five DAMs (Figure 11C): L-γ-glutamylcysteine, N-acetyl-L-aspartate, L-alanine, L-aspartate, and L-asparagine. In the purine metabolism pathway (Figure 12A), the expression levels of genes encoding *NDPK* and *APRT* were up-regulated under drought conditions (Figure 12B), leading to the accumulation of eight DAMs (Figure 12C).

## 3. Discussion

### 3.1. Effects of Drought Stress on Blueberry Physiology

Drought significantly inhibits plant growth and decreases fruit quality, with blueberry plants being particularly susceptible because of their shallow, hairless roots that limit water and nutrient uptake, especially in regions affected by drought. The leaf RWC is a crucial indicator of drought severity [18]. Plants that can tolerate drought stress have relatively high water levels and low dehydration rates, thereby maintaining metabolic functions [19]. In the current study involving ‘Emerald’ blueberry plants, the leaf water content decreased during treatments with different PEG-6000 concentrations; the decrease was greatest under severe drought conditions. A comparison of treatment effects indicated that high concentration–short duration and low concentration–long duration treatments resulted in similar drought stress conditions. The study findings provide valuable insights relevant to assessing and comparing drought tolerance among blueberry cultivars using PEG-6000.

### 3.2. Rapid Yeast-Based Screening of Blueberry Drought Tolerance

The complexity of the blueberry genetic background makes it difficult to identify drought tolerance-related genes. However, recently developed yeast libraries for high-throughput screening are useful for studying abiotic stress responses. Yeast grows rapidly and can be genetically modified relatively easily [20], making it an ideal model species for functionally characterizing plant genes [21]. Expressing plant cDNA in yeast enables researchers to directly analyze stress tolerance without the need for plant gene transfer. High-throughput sequencing facilitates the rapid identification of stress tolerance-related genes [22], while avoiding the limitations of transcriptome sequencing-based methods. Thus, yeast cDNA libraries offer a direct, efficient, and accurate alternative for isolating stress tolerance-related genes.

In this study, we established a yeast-based screening system to investigate blueberry root drought tolerance. We introduced drought-inducible blueberry genes into yeast cells to create an expression library. By screening this library under drought conditions, we identified yeast strains with enhanced drought tolerance. Additionally, on the basis of high-throughput sequencing, 1475 genes potentially related to drought tolerance were identified. A KEGG pathway enrichment analysis classified 77 key genes in six major metabolic pathways, whereas an untargeted metabolomic analysis detected 1749 DAMs in drought-tolerant yeast strains, including organic acids, amino acids and phenylpropanoids. Although a large number of tolerance genes can be identified using a yeast functional screening system, the satiation phenomenon of the yeast library may result in low abundance genes not being fully screened, thus affecting the comprehensiveness of the screening results. In future study, the sensitivity of the screening can be improved by performing fragment separation, optimizing the screening conditions and increasing the total size of the library to avoid the potential bias caused by library satiation.

Direct analyses of metabolites in plants can be challenging because of complex tissue structures and metabolite diversity [23]. Yeast is simpler than plants, but has common metabolic pathways, enabling it to be used for accurate analyses of plant metabolites [24]. Thus, an examination of yeast metabolomics may be useful for clarifying metabolite functions in plants. Our method involving the screening of transformed yeast cells-generated data useful for elucidating blueberry drought tolerance mechanisms. More specifically, key genes and metabolites for blueberry root drought tolerance were identified.

### 3.3. Drought-Induced Changes in Blueberry Root Gene Expression and Metabolic Pathways

An exposure to drought stress can substantially alter gene expression and metabolite accumulation in blueberry roots, with notable changes to genes and metabolites related to carbon metabolism, secondary metabolite biosynthesis, and nucleotide and amino acid metabolism. Carbon metabolism is essential for providing nutrients and energy under stress [25,26]. Our study results reflect the importance of inositol phosphate metabolism, the pentose phosphate pathway, and glycolysis/gluconeogenesis. GALM and PK (glycolysis/gluconeogenesis) and PIP5K (inositol phosphate metabolism) were identified as key enzymes for the synthesis of pyruvate and inositol, which are critical for ROS detoxification and energy production [27,28]. Pyruvate accumulation has been linked to increased stress tolerance [29].

Secondary metabolite biosynthesis enhances plant stress tolerance [30,31]. Our results are consistent with the findings of an earlier study in which terpenoid levels increased in response to severe drought stress [32]. The enrichment of FDPS genes and farnesylcysteine in the terpenoid biosynthesis pathway suggests they may contribute to osmotic stress tolerance [33]. Lignin synthesis mediated by CCR strengthens the cell wall and increases drought tolerance.

Purine metabolism is associated with salt tolerance [34]. In the current study, purine metabolism increased in drought-stressed blueberry roots, with inosine and 3′,5′-cyclic GMP potentially enhancing drought tolerance. The GST family in the glutathione pathway, which has been implicated in detoxification, may restrict the accumulation of harmful compounds under drought conditions [35,36]. Alanine, aspartate, and glutamate metabolism is also crucial for drought tolerance, with GLT1 and DAMs involved in the TCA cycle and amino acid biosynthesis, thereby promoting osmoregulation and antioxidant activities [37].

## 4. Materials and Methods

### 4.1. Plant Materials, Drought Stress Treatments, and Evaluation of Drought Tolerance

In this study, 3-month-old cuttings of the ‘Emerald’ blueberry cultivar served as experimental materials. Samples underwent a simulated drought stress treatment involving PEG-6000. Following a 7-day acclimation in half-strength Hoagland nutrient solution, plants were treated with various PEG-6000 concentrations (5%, 10%, 12.5%, 15%, 17.5%, and 20%) for 72 h. Plants grown in half-strength Hoagland nutrient solution alone served as the controls (i.e., CG). Subsequently, blueberry roots were harvested and stored at −80 °C.

To assess the effect of drought stress on blueberry cuttings, we examined the root condition and measured the leaf RWC. The leaf fresh weight (W_f_) was recorded. Leaves were then soaked in distilled water until fully saturated before being blotted to remove surface water and weighed to obtain their turgid weight (W_t_). Leaves were subsequently dried at 60–80 °C to a constant weight to determine their dry weight (W_d_). RWC was calculated using the following formula: RWC (%) = [(W_f_ − W_d_)/(W_t_ − W_d_)] × 100.

### 4.2. Construction of a Yeast Expression System

Establishing a high-quality yeast expression library comprising blueberry drought tolerance-related genes is critical for developing a yeast-based rapid screening system to elucidate the mechanisms regulating blueberry drought tolerance. Initially, an *E. coli* library containing cDNA associated with blueberry drought tolerance was constructed. Total RNA was extracted from treated roots and reverse-transcribed to synthesize high-quality cDNA, which was used as the template for PCR amplifications involving primers P1-F, P2-F, P3-F, and P4-R (Table S1). The amplified cDNA was purified to eliminate PCR by-products or contaminants, ensuring its suitability for further use. The purified cDNA was inserted into the pYES2 vector (i.e., a plasmid that can replicate independently). The resulting recombinant vector was inserted into *E. coli* TOP10 cells to establish a recombinant library. Positive *E. coli* colonies and cDNA length were verified by PCR using primers pYES2-F and pYES2-R (Table S1). Plasmids were extracted using a High-Concentration Plasmid Extraction Kit (TIANGEN Biotech Co., Ltd., Beijing, China) and stored at −20 °C as the library plasmids.

To construct a yeast expression library, the library plasmids were introduced into yeast strain *ycf1* cells, which were subsequently cultured on SG-Ura medium at 30 °C for 3–4 days to generate WG. Colonies were harvested, resuspended in YPDA solution (YPD + 25% glycerol), and pooled to create a cDNA library, which was designated as the drought stress library. Library quality was assessed using a Yeast Colony Rapid Detection Kit (Nanjing Ruiyuan Biotechnology Co., Ltd., Nanjing, China). Library capacity (cfu/mL) was calculated by dividing the number of clones by the coating volume and multiplying by the dilution factor; the total number of clones (cfu) was the product of the library capacity and the total volume of the yeast solution. Yeast cells harboring recombinant plasmids formed WG, whereas yeast cells carrying the empty pYES2 plasmid served as CG.

### 4.3. Functional Screening of the Yeast Expression System

To identify genes associated with blueberry drought tolerance using the yeast expression system, we screened yeast cells grown on a SG-Ura medium supplemented with PEG-4000. Cells that survived under drought stress conditions were selected to investigate the genes and mechanisms conferring drought tolerance. Initially, we established the PEG-4000 working concentration on the basis of a series of tests. Both WG and CG yeast cells were cultured for 3 days, after which they were added to plates containing solid SG-Ura medium supplemented with varying PEG-4000 concentrations (0, 50, 80, 100, and 120 mM). Following an additional 3-day incubation, WG cell growth was detected on medium containing specific PEG-4000 concentrations, whereas CG cells did not survive. The growth of WG cells on medium supplemented with PEG-4000 was attributed to the expression of drought tolerance-related blueberry genes, which enabled cells to maintain activities required for growth under stress. Of the tested concentrations, 100 mM PEG-4000 was selected for the subsequent functional screening of the yeast library. After clones were grown, 96 were randomly picked for a BLAST analysis. Following the removal of null and repetitive sequences, 21 different gene sequences were obtained. To test whether these screened genes can enhance yeast drought tolerance, the 21 positive clones were cultured in liquid SG-Ura medium containing 100 mM PEG-4000 for 3–4 days (with shaking). The cultures were then spotted on solid SG-Ura medium containing 100 mM PEG-4000 in plates. Yeast cells carrying the empty plasmid were used as the negative control.

For the analysis of regulatory genes and metabolites, three groups of cells were collected: Group A (CG cells) served as the control, Group B consisted of untreated WG cells, and Group C comprised WG cells that survived the drought treatment. Metabolites were extracted from the cells of all three groups for a metabolomic analysis conducted to identify metabolites associated with blueberry drought tolerance. Furthermore, plasmid DNA was extracted from Group C cells for a high-throughput sequencing analysis performed to identify genes involved in the drought stress response.

### 4.4. High-Throughput Sequencing and Identification of Potential Drought Tolerance-Related Genes

A PCR amplification was performed using primers pYES2-F and pYES2-R. The amplified products were fragmented (approximately 300 bp) to construct Illumina sequencing libraries using the NEBNext Ultra II DNA Library Prep Kit (NEB, Ipswich, MA, USA). Suitable libraries were sequenced using an Illumina HiSeq™ 2500 platform (Biomarker Technologies Corporation, Beijing, China). Clean reads were aligned to coding sequences in the Genome Database for Vaccinium using Burrows–Wheeler Aligner and Samtools algorithms. Coding sequences with mapped read counts of at least 20 were included in the library for further analyses. The GO enrichment analysis involved mapping all genes to terms in the GO database (http://www.geneontology.org/, accessed on 19 November 2023), whereas the KEGG pathway enrichment analysis was completed using the KEGG database (https://www.kegg.jp/, accessed on 19 November 2023). Specifically, hypergeometric testing was used to identify significantly enriched GO terms and KEGG pathways (*p* < 0.05) against the whole-genome background. The results of GO and KEGG enrichment analyses were visualized using OmicShare Tools (https://www.omicshare.com/tools/Home/Soft/, accessed on 23 November 2023) [38].

### 4.5. Metabolite Analysis

Metabolites in the cells from Groups A, B, and C were analyzed, with six replicates prepared for each group. Yeast cells were resuspended in a 400 µL extraction solution (MeOH:ACN, 1:1 (*v*/*v*)), vortexed for 30 s, sonicated in a 4 °C water bath for 10 min (this process was repeated three times), and then incubated at −40 °C for 1 h to precipitate proteins. The solution was centrifuged at 13,800× *g* for 15 min at 4 °C, after which the supernatant was transferred to a glass vial. A quality control sample was produced by pooling equal amounts of the supernatants from all samples. A metabolomic analysis was performed using a Vanquish UHPLC system (Thermo Fisher Scientific, Waltham, MA, USA) with a Waters ACQUITY UPLC BEH Amide column (2.1 mm × 50 mm, 1.7 µm), which was coupled to an Orbitrap Exploris 120 mass spectrometer (Thermo). The mobile phase comprised 25 mM ammonium acetate and 25 mM ammonia hydroxide in water (pH = 9.75) (A) and acetonitrile (B). The autosampler temperature was maintained at 4 °C, and the injection volume was 2 µL. MS/MS spectra were acquired in the information-dependent acquisition mode using Xcalibur software (Thermo) [39]. Metabolites were identified using the R package and BiotreeDB (v3.0) [40]. All identified metabolites were included in a PCA and an OPLS-DA. The criteria used to screen for DAMs were as follows: VIP > 1.0 and *p* < 0.05.

### 4.6. qRT-PCR Analysis

To examine the effect of drought stress on gene expression, ‘Emerald’ roots treated with 15% PEG-6000 were collected at various time points (0, 12, 24, 36, and 48 h). Total RNA was extracted from the collected roots according to the CTAB method and then reverse-transcribed to cDNA. Relative gene expression levels were quantified using the 2× SYBR Green qPCR Mix (High ROX) (AidLab Biotech, Beijing, China). Gene expression data were analyzed using the 2^−ΔΔCt^ method, with *VcGAPDH* serving as the reference gene [41]. The gene-specific primers used in this study are listed in Table S1. Gene expression was analyzed using three biological replicates and three technical replicates.

### 4.7. Statistical Analysis

Data were analyzed using SPSS 21.0 and Microsoft Office Excel 2016. All data were included in a one-way ANOVA with Duncan’s test, with *p* < 0.05 set as the threshold for significance.

## 5. Conclusions

**Figure 13:**
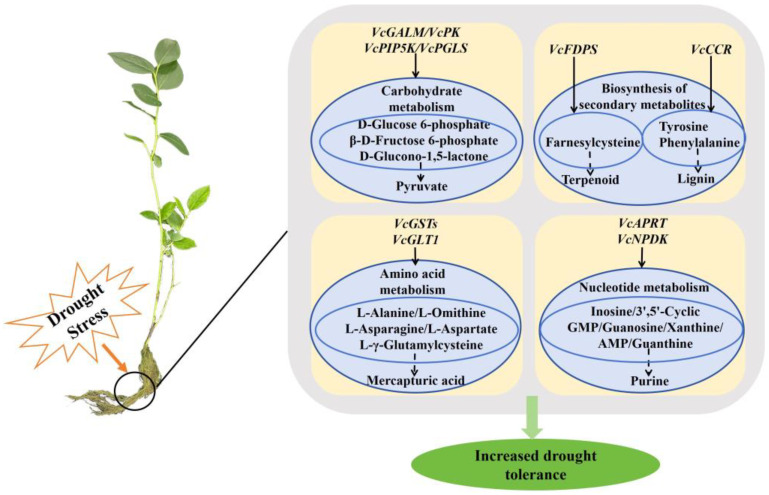
A conceptual model of key genes and metabolites affecting blueberry root drought resistance. This model identifies key genes and metabolites involved in carbon metabolism, secondary metabolite biosynthesis, and amino acid and nucleotide metabolism. The squares represent the genes, while the ellipses represent the metabolites. Solid arrows indicate the direct regulation of metabolites by genes, whereas dotted arrows suggest metabolites that are presumed to ultimately have functional roles.

In this study, we developed a yeast library comprising drought tolerance-related blueberry genes by extracting total RNA from the roots of drought-stressed ‘Emerald’ plants. We determined that 100 mM PEG-4000 was an appropriate concentration for screening drought-tolerant yeast strains. High-throughput sequencing and UHPLC-MS metabolite analyses identified 1475 genes and 1749 metabolites potentially associated with drought tolerance. Our integrated analysis indicated that blueberry roots respond to drought stress primarily by regulating the expression of key genes in carbon metabolism pathways (e.g., glycolysis/gluconeogenesis, pentose phosphate pathway, and inositol phosphate pathway), secondary metabolite biosynthesis pathways (e.g., terpenoid backbone and phenylpropanoid biosynthesis pathways), and nucleotide and amino acid metabolism pathways (e.g., purine metabolism, glutathione metabolism, and metabolism of alanine, aspartic acid, and glutamate). Genes encoding various enzymes, such as *GALM*, *PK*, *PGLS*, *PIP5K*, *FDPS*, *CCR*, *GST*, *GLT1*, *NDPK*, and *APRT*, were instrumental in enhancing the accumulation of metabolites involved in these processes, especially pyruvate, terpenoid, lignin, and glutathione, thereby strengthening the drought tolerance of blueberry roots (Figure 13). Thus, the methods used in this study effectively identified key genes and metabolites mediating the response of blueberry roots to drought stress. The study findings may be relevant to future investigations conducted to further elucidate the molecular mechanisms underlying blueberry drought tolerance.

## Data Availability

Data is contained within the article or Supplementary Materials.

## Supplementary Materials

The following supporting information can be downloaded at: https://www.mdpi.com/article/10.3390/plants13243528/s1, Table S1: Primers used in this study; Table S2: Details regarding candidate genes; Table S3: KEGG functional enrichment analysis of drought tolerance-related genes; Figure S1: Construction and analysis of the primary library; Figure S2: Yeast colony PCR; Figure S3: Score scatter plot of the PCA model for Groups A, B, and C; Figure S4: Validation of the OPLS-DA model; Figure S5: Heat map of all metabolites.
