# Supplementary material for: Unraveling the Molecular Mechanisms of Blueberry Root Drought Tolerance Through Yeast Functional Screening and Metabolomic Profiling
Source: Plants (Basel). 2024 Dec 17;13(24):3528. doi: 10.3390/plants13243528 (PMC11678528; doi:10.3390/plants13243528)
Supplement: Supplementary file 1 [file plants-13-03528-s001.zip › plants-3357985-supplementary materials.pdf]

Supplementary Materials

Legends of Supplementary Tables and Figures

Figure S1. Construction and analysis of the primary library

Figure S2. Yeast colony PCR

Figure S3. Score scatter plot of the PCA model for Groups A, B and C

Table S1. Validation of the OPLS-DA model

Table S2. Heat map of all metabolites

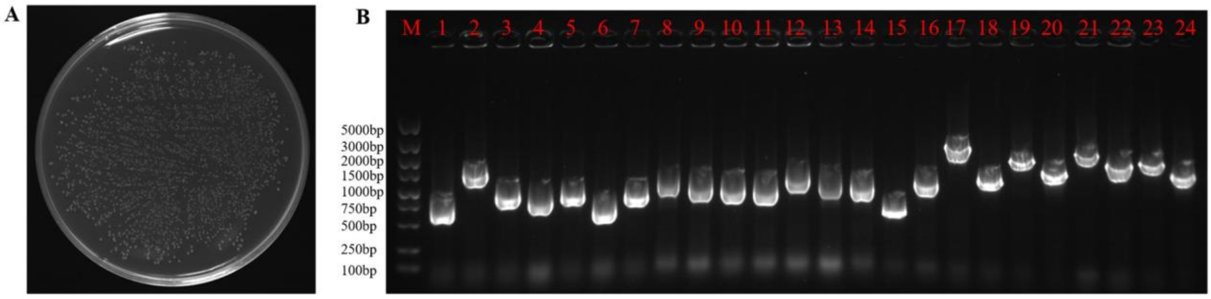

Figure S1: Construction and analysis of the primary library.
A): E. coli colony count. E. coli colony plates were photographed after 12 h, the dilution ratio was 10000, and the number of plate clones was about 2456. B) Library quality evaluation. E. coli colony PCR, M: Maker, 1-24:24 clones were randomly selected for PCR, and electrophoresis was performed after PCR.

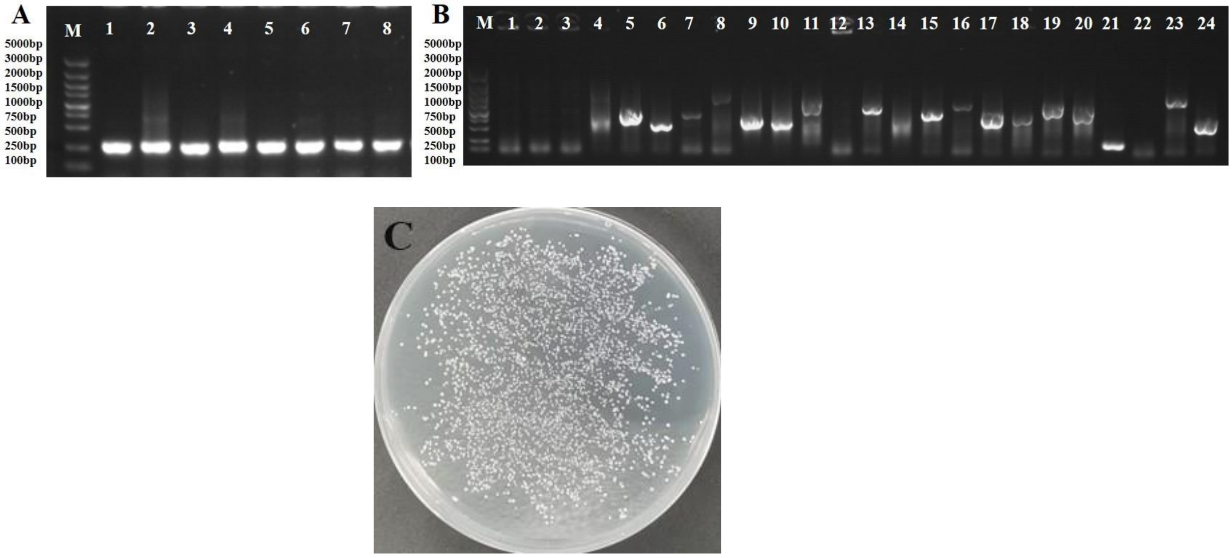

Figure S2: Yeast colony PCR
A): PCR of empty plasmid pYES2 clones. B): PCR of blueberry library clones. C): Yeast stock solution coated after 10,000-fold dilution.

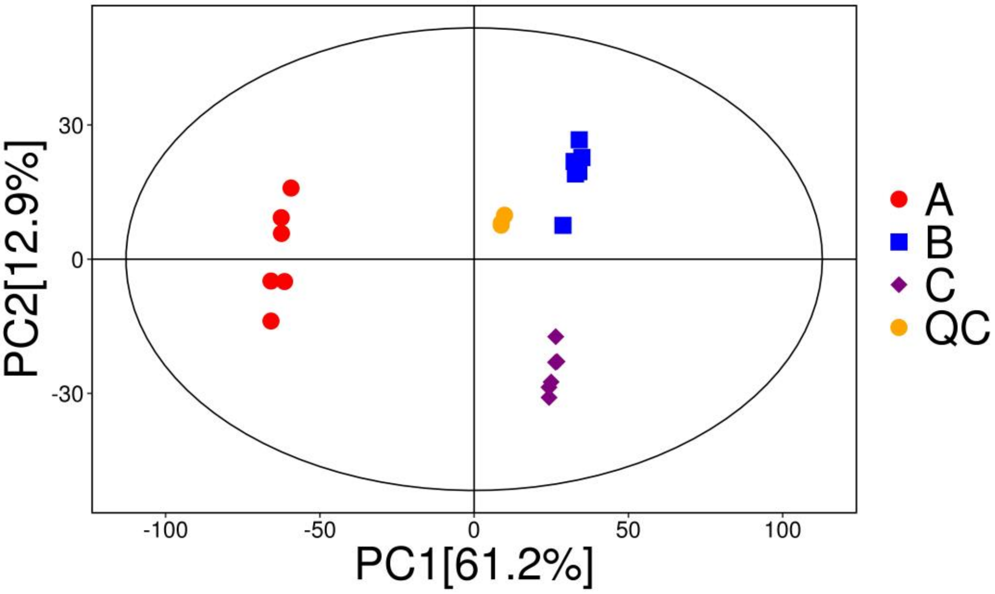

Figure S3: Score scatter plot of the PCA model for Groups A, B and C.

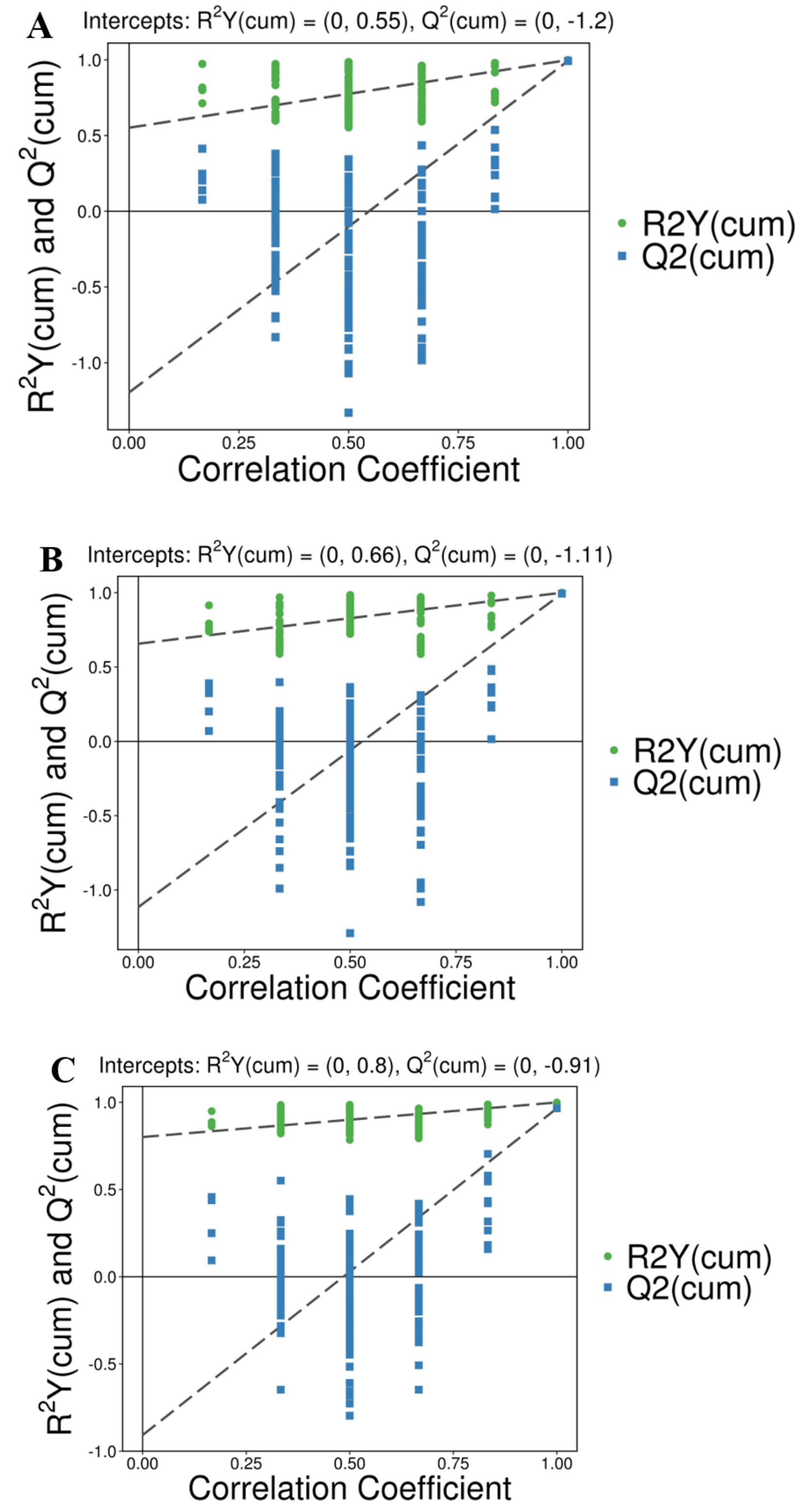

Figure S4: Validation of the OPLS-DA model. A): A and B. B): A and C. C): B and C.

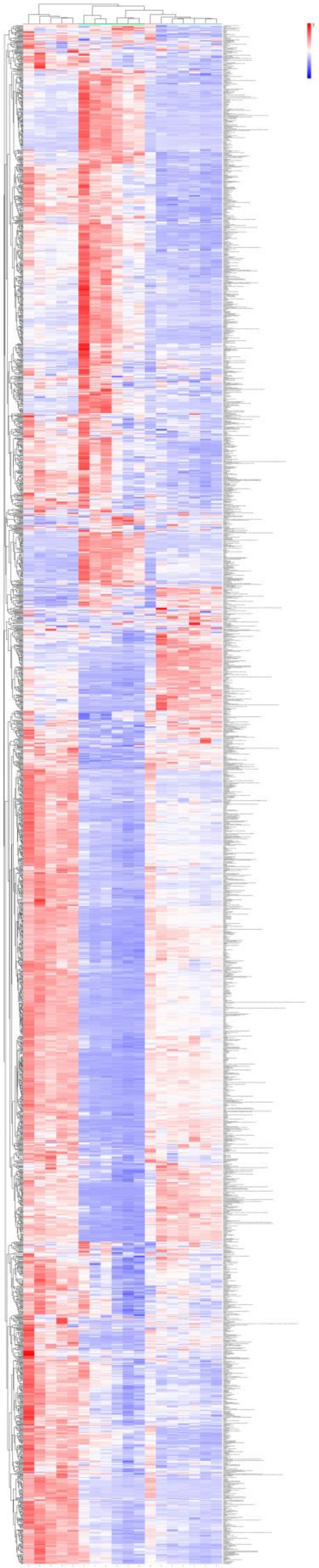

Figure S5: Heat map of all metabolites.
